# Special Issue: Latest Research on Plant Genomics and Genome Editing

**DOI:** 10.3390/ijms26209946

**Published:** 2025-10-13

**Authors:** Fabrizio Carbone

**Affiliations:** Research Center for Olive, Fruit and Citrus Crops, Council for Agricultural Research and Economics (CREA), Via Settimio Severo 83, 87036 Rende, CS, Italy; fabrizio.carbone@crea.gov.it

## 1. Introduction

Over the past ten years, plant science has undergone a remarkable transformation driven by the convergence of next-generation sequencing, increasingly sophisticated bioinformatics tools, and the rise of targeted genome editing platforms. These advances have significantly deepened our understanding of plant biology at multiple levels—from gene structure and function to regulatory networks and epigenetic landscapes.

In parallel, the escalating demands posed by global climate change, declining biodiversity, and a rapidly growing human population have placed unprecedented pressure on agricultural systems. These challenges highlight the urgent need for innovative and sustainable strategies to improve crop performance, resilience, and nutritional value.

This Special Issue, “Latest Research on Plant Genomics and Genome Editing”, brings together a diverse collection of original research articles and reviews that reflect the current momentum in this rapidly evolving field. The studies featured here encompass a wide array of topics, including transcriptional regulation, epigenetic remodeling, non-coding RNA function, stress signaling, and CRISPR-based molecular tools.

In this editorial, we aim to highlight the main contributions included in this issue, explore their relevance within the broader scientific context, and outline the key directions shaping the future of plant genomics and genome editing.

## 2. Overview of the Special Issue Contributions

The research featured in this Special Issue reflects the vibrant diversity of approaches currently used to investigate and manipulate plant genomes. Across different species and biological systems, the selected papers illustrate how molecular tools, computational resources, and genome editing technologies are converging to advance both basic understanding and practical applications in plant science. Several thematic clusters can be identified:

### 2.1. Transcription Factors and Regulatory Gene Networks

Transcription factors (TFs) are fundamental components of gene expression control, enabling plants to fine-tune developmental processes and respond dynamically to environmental stressors. In this collection, four contributions provide genome-wide insights into TF families relevant to stress adaptation, development and secondary metabolism regulation:

- A study focusing on WRKY TFs in various grapevine (*Vitis vinifera*) varieties characterizes their structural diversity, evolutionary patterns, and stress-responsive expression profiles. The identification of candidate genes potentially involved in biotic and abiotic stress tolerance lays the groundwork for future functional validation and breeding efforts [1].
- In sugar beet (*Beta vulgaris*), the functional roles of three SQUAMOSA Promoter Binding Protein-Like (SPL) genes—SPL6, SPL7, and SPL9—were investigated. Experimental data indicate their involvement in enhancing drought tolerance through improved photosynthesis and reactive oxygen species (ROS) scavenging. These findings suggest a promising avenue for improving crop resilience in saline or arid environments [2].
- In Poplar (*Populus alba* × *Populus glandulosa* and *Populus simonii* × *Populus nigra*), the unique VeA transcription factor family associated with secondary metabolism and the ethylene-insensitive3/ethylene-insensitive3-like (EIN3/EIL) TF family were characterized. Based on phylogenetics, genomic distribution, gene structure and conserved motif, promoter binding site, and expression profiles analysis, the first study comprehensively and systematically provides a basis for the biological function and regulatory mechanism of the VeA gene family in regulating secondary metabolism in poplar [3]. In the second study, findings suggest that the EIN3/EIL gene family may play a significant role in the growth and development of poplar [4].

### 2.2. Epigenetic Regulation via DNA Demethylation

Epigenetic mechanisms such as DNA methylation and demethylation are critical for regulating plant gene expression in response to environmental stimuli. One study conducted in Foxtail millet (*Setaria italica*) identified members of the DNA demethylase (DML) gene family and evaluated their differential transcriptional responses under salt, cold, and drought conditions [5]. The observed stress-responsive expression patterns suggest that dynamic methylation remodeling may play a significant role in abiotic stress adaptation in this drought-tolerant crop species.

### 2.3. Non-Coding RNA-Mediated Regulation Under Cold Stress

Long non-coding RNAs (lncRNAs) and microRNAs (miRNAs) are now recognized as key layers of post-transcriptional regulation in plants. In Rice (*Oryza sativa* L.), a detailed transcriptomic analysis under cold stress revealed a complex network involving lncRNAs, miRNAs, and their mRNA targets [6]. The identification of hub lncRNAs and their associated regulatory interactions provides new candidate molecules for dissecting cold tolerance mechanisms and for potential use in targeted breeding strategies.

### 2.4. Circadian Gene Networks and Light-Regulated Transcriptomics in Olive

Light is a central environmental cue affecting plant development, metabolism, and flowering. A transcriptomic study in olive (*Olea europaea* L.) explored the impact of continuous light exposure on the circadian clock and associated gene networks [7]. Differentially expressed genes involved in photoreception, secondary metabolism, and fatty acid biosynthesis were identified, alongside shifts in the expression of key clock components. These results suggest that continuous photoperiods could influence flowering timing, development and oil biosynthesis in perennial crops.

### 2.5. Functional Peptides in Salt Stress Tolerance

Secreted peptides have recently emerged as important regulators of abiotic stress responses. One study investigated the functional role of a small peptide, SUBPEP3, in Pear (*Pyrus betulifolia*). Transgenic lines overexpressing this peptide exhibited improved salt stress tolerance, as evidenced by increased activity of antioxidant enzymes and accumulation of osmoprotective compounds [8]. These findings demonstrate the potential of peptide-based biotechnological strategies in enhancing stress resilience in fruit crops.

### 2.6. Expanding the CRISPR Toolkit: Beyond Genome Editing

While CRISPR/Cas systems were initially developed for targeted gene disruption, their versatility has since expanded dramatically. A comprehensive review [9] in this Special Issue outlines the full spectrum of CRISPR-based applications now available for plant biotechnology, including the following:

- Base editors and prime editors for precision DNA modifications.
- Transcriptional regulation systems for gene activation or repression (CRISPRa/i).
- Epigenetic editing tools (e.g., dCas9 fused with TET or DNMT domains).
- RNA-targeting platforms such as Cas13 variants.
- Diagnostic technologies like SHERLOCK.

These innovations highlight the growing capacity of CRISPR-based systems not only to alter DNA sequences, but also to modulate gene expression, chromatin accessibility, and even RNA metabolism—all with increasing accuracy and minimal off-target effects. This expanded toolkit opens new avenues for both functional genomics and next-generation crop improvement.

## 3. Current Landscape and Strategic Directions in Plant Genomics and Genome Editing

Plant genomics and genome editing have advanced rapidly, yet practical applications face ongoing challenges as the field shifts from proof-of-concept studies to real-world, large-scale agricultural solutions. Early efforts centered on gene knockouts using CRISPR/Cas9 have evolved into precision techniques like base and prime editing, cis-regulatory modifications, and conditional expression systems—aimed at fine-tuning gene function with greater accuracy and fewer side effects. However, efficient and species-independent delivery methods, particularly for complex or polyploid genomes, remain a major hurdle. In parallel, multi-omics approaches—combining transcriptomics, epigenomics, proteomics, and metabolomics—are essential for decoding complex traits, though data integration and interpretation require advanced computational tools and standardized frameworks. The emergence of pangenomics has uncovered structural variation and untapped genetic diversity critical for trait improvement, though building and maintaining such resources is technically demanding. Regulatory and ethical landscapes are equally complex: inconsistent international policies and public skepticism hinder global collaboration and adoption. Looking forward, strategic priorities include advancing transformation and DNA-free delivery systems (e.g., nanocarriers, RNPs), embedding genome editing within precision agriculture frameworks, and employing multiplex editing for climate-resilient trait stacking. Simultaneously, public engagement, open-access resources, and inclusive policymaking are essential to ensure equitable access and societal trust. Ultimately, the integration of plant genomics with synthetic biology, AI-driven analytics, and stakeholder participation offers a powerful route toward resilient, sustainable crops that meet global food and environmental demands.

## 4. Concluding Remarks

This Special Issue encapsulates the dynamic and multidimensional progress occurring at the interface of plant genomics and genome editing. The highlighted studies collectively deepen our understanding of regulatory networks, epigenetic modifications, and novel molecular tools while illustrating the expanding potential of CRISPR-based technologies for precise and versatile plant improvement. As the field advances, a shift from single-gene edits to holistic, multi-layered approaches will be critical for addressing the complex challenges posed by climate change, food security, and environmental sustainability. Integrative frameworks that combine genomics, phenomics, synthetic biology, and computational modeling, coupled with transparent public engagement and ethical governance, will drive the next wave of innovation. Looking forward, interdisciplinary collaboration and international cooperation will be indispensable to harness the full potential of plant genome editing technologies. Through shared knowledge, responsible stewardship, and inclusive policies, the scientific community can deliver resilient, nutritious, and sustainable crops that meet the demands of a growing global population. We extend our sincere gratitude to all contributors and reviewers whose expertise and dedication have shaped this Special Issue into a timely and valuable resource. It is our hope that these collective insights will inspire further research and innovation in plant genomics and genome editing, ultimately fostering a sustainable agricultural future.
